# Necrotizing autoimmune myositis following coronavirus disease 2019 infection: a case report

**DOI:** 10.1186/s13256-022-03680-5

**Published:** 2022-12-27

**Authors:** Maryam Loghman, Ehsan Rahmanian, Majid Alikhani, Hiva Saffar, Sara Beikmohamadi Hezaveh, Mohammad Nekooeian, Reza Shahriarirad, Seyedeh Tahereh Faezi

**Affiliations:** 1 Department of Internal Medicine, School of Medicine, Rheumatology Research Center, Shariati Hospital, Tehran University of Medical Sciences, Tehran, Iran; 2 Department of Rheumatology, Hormozgan University of Medical Sciences, Bandar Abbas, Iran; 3 Department of Pathology, Shariati Hospital, Tehran University of Medical Sciences, Tehran, Iran; 4 Resident of Neurology, Department of Neurology, Shariati Hospital, Tehran University of Medical Sciences, Tehran, Iran; 5 Health and System Research Center, Shiraz University of Medical Sciences, Shiraz, Iran; 6 Thoracic and Vascular Surgery Research Center, Shiraz University of Medical Sciences, Shiraz, Iran; 7 School of Medicine, Shiraz University of Medical Sciences, Shiraz, Iran

**Keywords:** COVID-19, Myalgia, Myositis, SARS-CoV-2, Muscle weakness, Case report

## Abstract

**Background:**

Severe acute respiratory syndrome coronavirus 2 may be associated with late-onset necrotizing myositis, mimicking autoimmune inflammatory myositis; however, the exact underlying pathogenesis of severe acute respiratory syndrome coronavirus 2-induced myositis is still unclear.

**Case Presentation:**

Herein, we report a rare case of necrotizing autoimmune myositis in a 67-year-old middle eastern male following coronavirus disease 2019 infection, who presented with muscle weakness. The patient had positive anti-NXP2. The diagnosis of necrotizing autoimmune myositis was made according to muscle weakness, increased liver enzymes, electromyography and nerve conduction velocity results, and muscle biopsy. The patient underwent a full malignancy evaluation, which was unremarkable, and was discharged in relatively well condition with a daily dose of 1 mg/kg prednisolone and azathioprine 150 mg (2 mg/kg).

**Conclusion:**

Our report highlights the already known possible protracted sequence of coronavirus disease 2019 infection and the potential for delayed-onset necrotizing myositis.

## Introduction

Since the beginning of the coronavirus disease 2019 (COVID-19) pandemic in 2019, a wide variety of signs and symptoms have been associated with this multisystem viral disease. Myalgia is typically reported in symptomatic patients alongside other symptoms such as fever, cough, and shortness of breath [1]; however, in relatively rare cases, patients may develop COVID-19-related rhabdomyolysis or myositis [2].

Necrotizing autoimmune myositis (NAM) is a variant of idiopathic inflammatory myopathies (IIM) that has been reported in a few cases following COVID-19 infection [3]. Herein, we report a rare case of NAM in a 67-year-old man following COVID-19 infection.

## Case report

### History and presentation

The patient was a 67-year-old Middle-Eastern man who came to the emergency ward with progressive bilateral lower extremity muscle weakness and myalgia. The patient had no drug history and no significant past medical history or history of contact with toxins. Fifteen days prior to the onset of muscle weakness, the patient had presented with fever, rhinorrhea, and mild shortness of breath, and with a positive nasal swab PCR test, had a diagnosis of severe acute respiratory syndrome coronavirus 2 (SARS-CoV-2) pneumonia, for which he was treated as an outpatient, with desirable recovery after 15 days. However, following the resolution of COVID-19 symptoms, the patient started to develop myalgia and bilateral lower extremity proximal weakness. Due to augmentation of his lower extremity weakness and development of upper extremity proximal weakness, he was admitted to the hospital for further evaluation. In the physical examination, proximal muscle weakness was noted in the upper and lower extremities (power grade 4/5 and 3/5, respectively), with normal tone and deep tendon reflexes. The patient had no skin lesions or rashes. The patient also reported no muscle tenderness in our examination. The rest of the examination was normal.

### Investigations

**Fig. 1:**
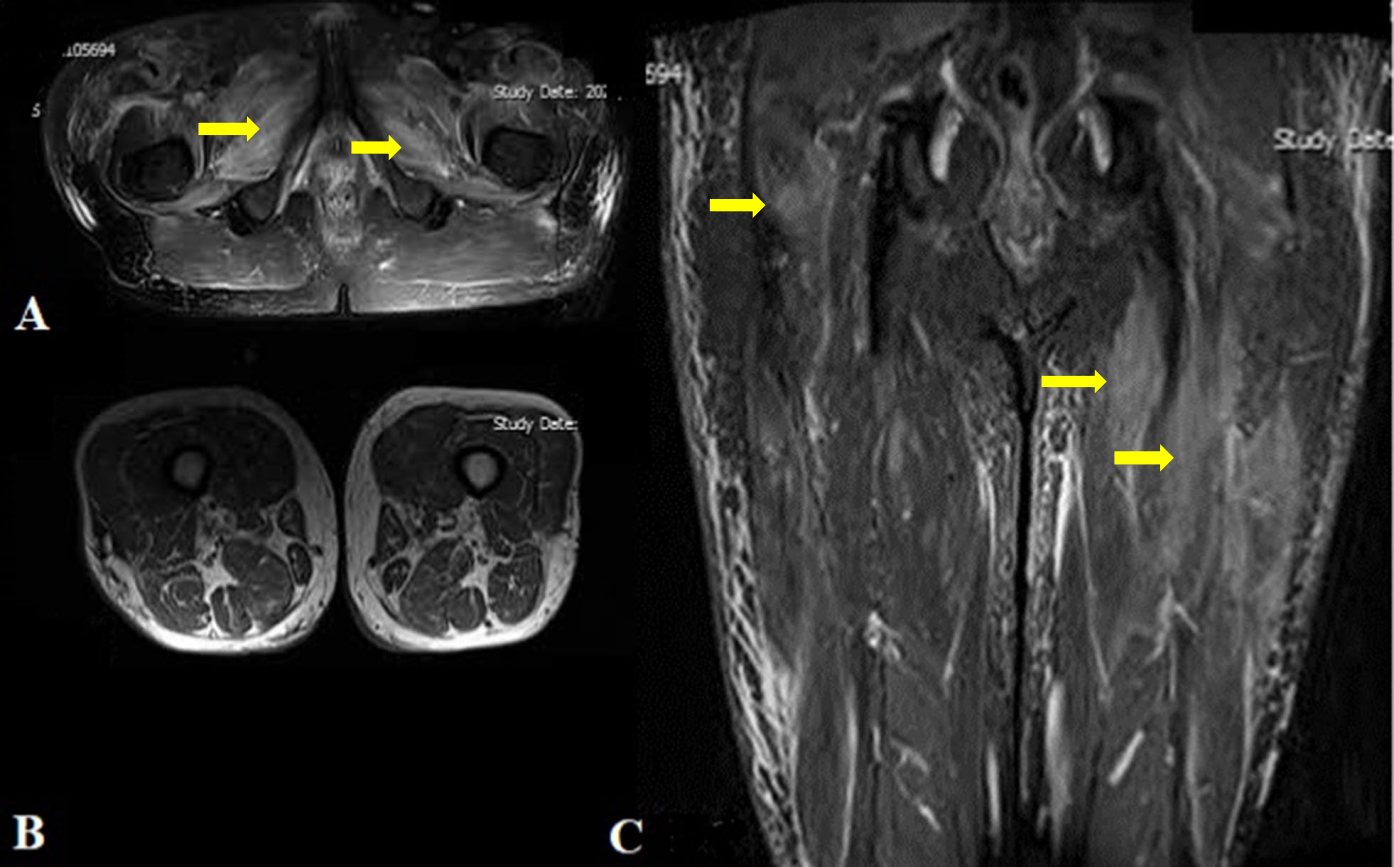
Bilateral thigh magnetic resonance imaging (without gadolinium contrast medium) T1 and T2 in axial and coronal view demonstrating diffuse edema within all muscle compartments of the thigh with notable intra- and perimuscular edema, suggestive of myositis; **A** axial T2; **B** axial T1; **C** coronal T1 (indicated with yellow pointer)

The initial laboratory tests detected elevated muscle enzymes including creatine phosphokinase (CPK): 12,000 (normal: 38–174 U/L); lactate dehydrogenase (LDH): 757 (normal: 130–240 U/L)]; and aldolase: 11 (normal: 8.5 U/L). Procalcitonin was in the normal range. Also, 25-hydroxy vitamin D test levels were normal, at 40 ng/mL. Other laboratory data included a white blood count (WBC) of 8.83 × 10^3^ µl), hemoglobin of 14.2 g/dL, erythrocyte sedimentation rate (ESR) of 9 mm/hour (normal: < 20 mm/hour), C-reactive protein of < 2 mg/L (normal: ≤ 6 mg/L), creatinine of 1 mg/dL, blood urea of 32 mg/dL, sodium (Na) 140 mEq/L, potassium (K) 4.4 mEq/L, aspartate aminotransferase (AST) of 25 IU/L (normal: 15–40 IU/L), alanine transaminase (ALT) 26 IU/L (normal: ≤ 41 IU/L), and alkaline phosphatase 121 IU/L (normal 120–450 IU/L). Bilateral thigh magnetic resonance imaging (MRI) axial and coronal view (T1 and T2; Short Tau Inversion Recovery) showed diffuse edema within all muscle compartments of the thigh with notable intra- and perimuscular edema, suggestive of myositis. (Fig. 1).

**Fig. 2:**
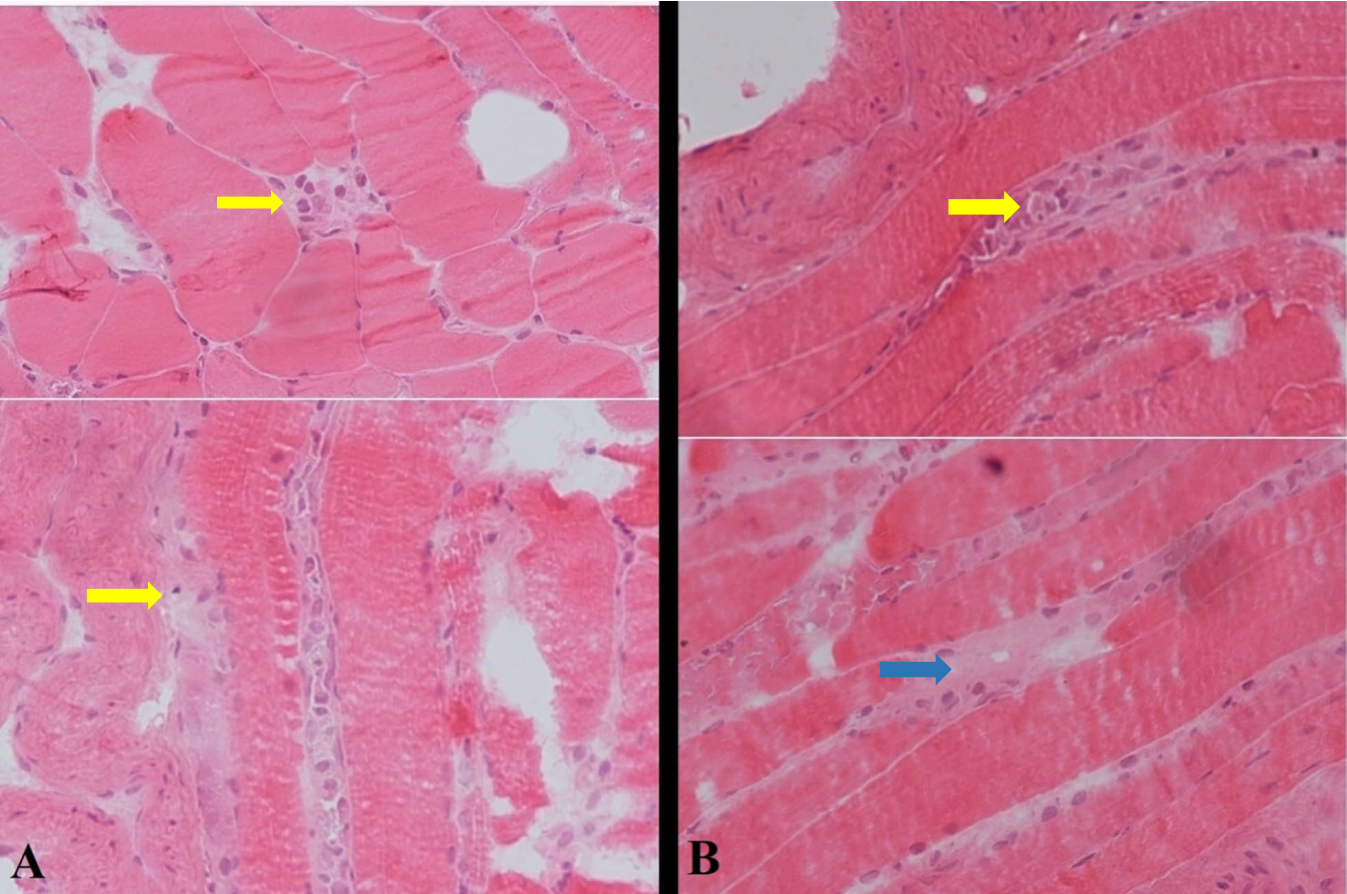
**A** Transection of muscle biopsy showing some pale necrotic fibers with evidence of myophagocytosis (indicated with yellow pointer) (H&E; ×400) **B** Longitudinal section of muscle fibers with variation in size, including some pale necrotic fibers (blue arrow) with evidence of myophagocytosis (indicated with yellow pointer) (H&E; ×400)

Electromyography (EMG) and nerve conduction velocity (NCV) findings were suggestive of irritable myopathy in several proximal muscles, including rectus femoris, iliopsoas, and deltoid. Additionally, in a left deltoid muscle biopsy, muscle necrosis was reported in the absence of inflammation, in favor of necrotizing autoimmune myositis (Fig. 2). In cryopreserved biopsy from deltoid muscle, variation in fiber size was evident, with presence of some pale necrotic fibers with evidence of myophagocytosis associated with some regenerating fibers. Inflammation was absent. On trichrome stain, no apparent fibrosis was seen. Based on the overall histomorphology and clinical and laboratory findings, the diagnosis of immune-mediated necrotizing myopathy was established. (Fig. 2).

**Fig. 3:**
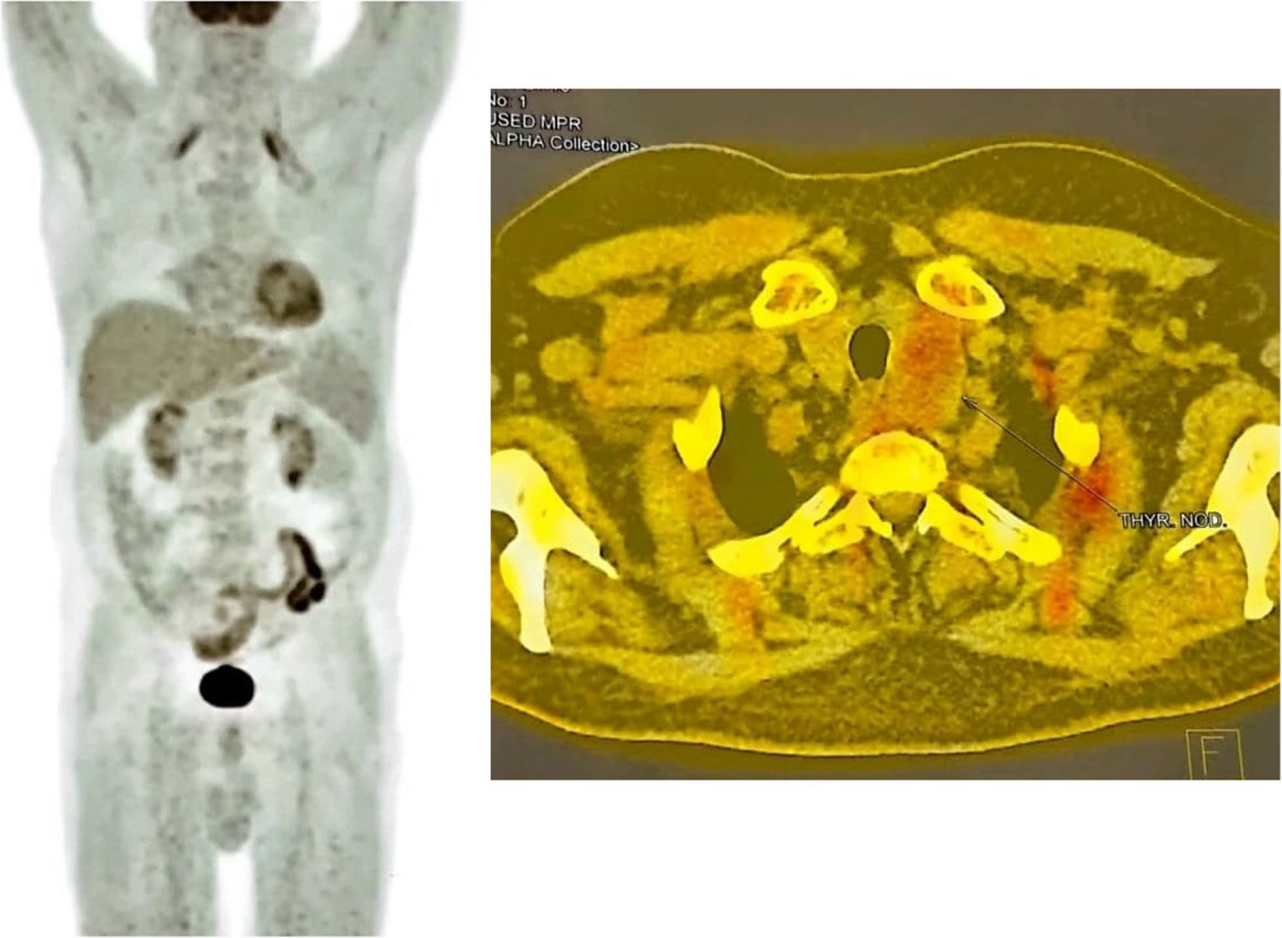
Fluorodeoxyglucose-positron emission tomography scan of a 67-year-old male patient with necrotizing autoimmune myositis following coronavirus disease 2019 infection

**Fig. 4:**
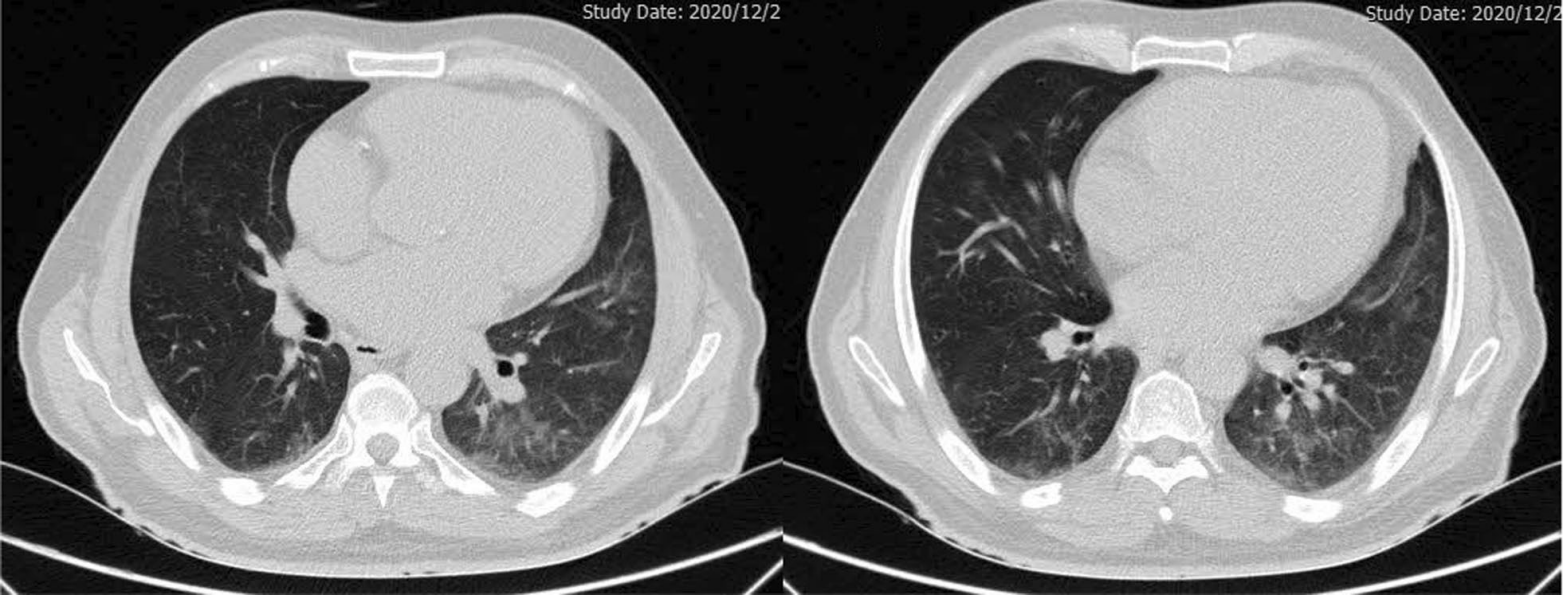
Chest high resolution computed tomography scan (without Contrast, Axial view) of a 67-year-old male patient with necrotizing autoimmune myositis following COVID-19 infection, demonstrating ground glass opacity in basal parts of the lung along with mild pericardial effusion

Among all myositis-related autoantibodies such as anti-3-hydroxy-3-methylglutaryl-coenzyme A reductase (HMGCR), anti-signal recognition particle (SRP), anti-Jo-1, and anti-NXP2, only anti-NXP2 autoantibodies were positive, which prompted us to search for underlying malignant processes. The patient underwent a malignancy workup by abdomen and pelvis ultrasound, chest CT scan, endoscopy and colonoscopy, prostate-specific antigen (PSA), and ear, nose, and throat (ENT) examination, which were all unremarkable. Based on the positive results of NXP2 and to rule out malignancy, a fluorodeoxyglucose (FDG)-positron emission tomography (PET) scan was performed, which demonstrated a 35 × 28 mm left thyroidal lobe nodule (Fig. 3). A thyroid biopsy confirmed its unmalignant nature and thyroid-stimulating hormone (TSH) levels were also normal. Cardiac echocardiography, electrocardiography, and lung spirometry were also normal. Chest high-resolution computed tomography (HRCT) also showed bilateral multifocal patchy ground-glass opacities with dominant peripheral distribution, which were attributable to previous COVID-19 pneumonia. (Fig. 4).

Since myositis could emerge in the context of some connective tissue disorders, testing for some other laboratory markers, including antinuclear antibodies (ANA), anti-smooth muscle antibodies (ASMA), perinuclear antineutrophil cytoplasmic antibodies (p-ANCA), cytoplasmic antineutrophil cytoplasmic autoantibody (C-ANCA), anticyclic citrullinated peptide (anti-CCP), rheumatoid factor (RF), anti-double-stranded DNA (anti-ds DNA), complement component 3 (C3), complement component 4 (C4), and anti-Ro were done, which were all in the normal range. Thus, a diagnosis of necrotizing autoimmune myositis, possibly complicating COVID-19, was made after ruling out other causes for NAM by appropriate tests.

### Treatment and follow-up visit

For treatment, prednisolone 1 mg/kg orally was administered. CPK levels started to fall after the initiation of steroids and were 107 IU/mL at the time of discharge. Resolution of ground-glass opacity of the lungs was observed after three months from his discharge. The prednisolone dose was decreased gradually to 10 mg, 1 year after diagnosis, and azathioprine was continued with an initial dose of 150 mg/day, which was decreased to 100 mg/day. The patient’s muscle force was resolved to normal after 1 year of follow-up. After confirming no inflammation, we reduced the prednisolone dose to an eventual 5 mg daily, along with 50 mg/day azathioprine. The patient is still relatively well after 15 months follow-up with improved laboratory examinations and muscle power in all extremities. The latest laboratory data were as follows: CPK: 115 U/L; LDH: 478 U/L; WBC: 9.02 × 10^3^/µl); hemoglobin: 13.9 g/dL; AST: 25 IU/L; ALT: 23 IU/L; alkaline phosphatase (ALK): 134 IU/L; K: 4.1 mEq/L; ESR: 5 mm/hr; CRP < 2; vitamin D: 30 ng/mL; creatinine: 1.19 mg/dL; blood urea: 42 mg/dL; and Na: 139 mEq/L.

## Discussion

IIM are a group of immune-mediated diseases that, despite distinctive clinical features, present with muscle inflammation and progressive muscle weakness [4]. NAM is a major subgroup of IIM that usually presents with subacute proximal muscle weakness, dysphagia, and dyspnea [5]. Known triggers of this disease are a number of drugs such as statins, infection, and malignancies [6].

NAM has been reported in the context of malignant, statin-induced infections, in which some are common in our region [7–13]; however, there are limited reports regarding this entity following COVID-19 infection [3]. Various medications and treatments have been proposed for COVID-19 [14–17], which along with diagnosis, hospital policies, and high vaccination rates, have increased the control over the disease outbreak [18–22]. However, the long-term adverse effects of patients affected by this disease are still unknown, and reports in this regard are limited [23–25]. Although NAM has been reported following various infective pathogens, reports regarding post-SARS-CoV-2 infection are limited [3, 26]. Since the COVID-19 outbreak has caused a major public health concern and affected many patients [27–30], the possibility of such complications among survivors should be taken into consideration by physicians and rheumatology specialists. Increasing public and healthcare worker knowledge regarding this issue can assist in timely diagnosis and management of the disease [31].

In most cases, SRP or HMGCR autoantibodies are detected in affected patients [4]; in our case, however, the patient had a negative history of statin use, and SRP and HMGCR autoantibodies were not detected. Vitamin D deficiency was ruled out based on normal 25-hydroxy vitamin D levels, and the patient reported no suspicious history of toxin-mediated NAM. All of the malignancy workups were negative. Another distinctive feature of NAM is the markedly elevated CK level that usually ranges in thousands as seen in our patient, and helps to narrow down the diagnosis and carry out further workups [32].

Another myositis-specific autoantibody (MSA) that was tested for in our patient was NXP-2, which is a major MSA in juvenile dermatomyositis. Studies have shown that this autoantibody is closely related to malignancy in adults and patients who were diagnosed with cancer around the time the antibody was detected [33]. Hence, as anti-NXP2 autoantibodies are often associated with paraneoplastic syndrome myositis, an extensive workup was done, but no sign of malignancy was detected. A study by Lee et al. [34] also reported development of anti-NXP2 dermatomyositis following Comirnaty COVID-19 vaccination, and an initial presentation of myalgia, crusted eruptions, and also elevated CPK levels, which was eventually treated with pulsed intravenous methylprednisolone followed by 1 mg/kg prednisolone. In our case, although anti-NXP2 was positive, we performed a thorough evaluation for findings in favor of malignancy, which were all unremarkable.

When myositis is the most probable diagnosis, a physician should have a thorough approach as this presentation has a wide-ranging differential diagnosis from metabolic etiologies such as hypothyroidism, to paraneoplastic-related myositis. Moreover, some viruses such as coxsackievirus and human T-lymphotropic virus can cause myositis by direct invasion of muscles as well as delayed immune injury.

Viral infections, especially those which infect the respiratory system, are believed to be another cause of inflammatory myopathies [6]. Different mechanisms are proposed for muscle injury after viral infection. A study on inflammatory myositis and COVID-19 suggested an association between COVID-19 and NAM, and a possible increase of NAM during the pandemic which may have been overlooked [4].

A helpful tool in diagnosis of inflammation is MRI [35]. In MRI of affected muscles in patients with NAM, generalized muscle edema, atrophy, and fatty replacement is seen. However, the severity and pattern are different in SRP and HMGCR autoantibody-positive patients. In anti-SRP-positive patients, the findings are more prominent and severe muscle edema is observed in the vastus lateralis, rectus femoris, biceps femoris, and adductor magnus [35, 36].

Few cases of NAM have been reported in COVID-19 positive patients. Reggio et al. reported a case of a COVID-19 patient who developed new-onset lower extremity weakness 16 days after the diagnosis, with elevated CK and impaired thyroid panel [37]. The CK raised after the discontinuation of statin medication, so with further workups, including muscle biopsy, the diagnosis of necrotizing myopathy and thyroiditis was made. A different report of a 51-year-old man, made by Lokineni et al., who presented with increased CK and lower extremity weakness, 3 month after COVID-19 infection. A diagnosis of necrotizing myositis was established after muscle biopsy [3]. Another similar study reported a case in which the patient presented with worsening dyspnea and elevated CK 1 month after COVID-19 infection, but the lower extremity weakness started after 3 months [26]. These studies are all similar to our report, in which the patient presented with increased CK and acute lower extremity weakness weeks after the initial COVID-19 infection. However, there are also reports of myositis as the first presentations of COVID-19 [38].

## Conclusions

SARS-CoV-2 may be associated with necrotizing myositis, mimicking autoimmune inflammatory myositis; however, the exact underlying pathogenesis of SARS-CoV-2-induced myositis is still unclear. Although anti-NXP2 was positive in our case, there were no signs of malignancies in our evaluations. Our report highlights the already known possible protracted sequence of COVID-19 infection and the potential for necrotizing myositis necessitating long-term immunosuppression.

## Data Availability

All data regarding this study has been reported in the manuscript. Please contact the corresponding author if you are interested in any further information.

## Abbreviations

COVID-19: Coronavirus disease 2019
CT: Computed tomography
HRCT: High-resolution computed tomography
EMG-NCV: Electromyography and nerve conduction velocity
FDG-PET: Fluorodeoxyglucose-positron emission tomography
IIM: Idiopathic inflammatory myopathies
MRI: Magnetic resonance imaging
NAM: Necrotizing autoimmune myositis
PCR: Polymerase chain reaction
